# Role of HNF6 in liver homeostasis and pathophysiology

**DOI:** 10.1186/s10020-025-01105-9

**Published:** 2025-02-05

**Authors:** Miaomiao Tian, Weizhen Gao, Shujun Ma, Huiling Cao, Yu Zhang, Fuxiang An, Jianni Qi, Zhen Yang

**Affiliations:** 1 Shandong Provincial Hospital Affiliated to Shandong First Medical University, Jinan, 250021 Shandong P. R. China; 2 Shandong Provincial Engineering and Technological Research Center for Liver Diseases Prevention and Control, Jinan, 250021 Shandong P. R. China

**Keywords:** HNF6, Epigenetic modifications, Metabolism, Liver pathophysiology, Transcription factor

## Abstract

**Background:**

Hepatocyte nuclear factor 6 (HNF6), a member of the HNF family, contains single cleft and homologous domains, which form a DNA-binding region that targets the promoter regions of genes that bind to liver-specific genes and regulate their expression. Furthermore, HNF6 is highly expressed as an HNF in the liver.

**Main body:**

HNF6 regulates not only the formation of the liver but also the proliferation and differentiation of hepatocytes. Additionally, HNF6 controls the migration and adhesion of hepatocellular carcinoma cells and plays a significant role in liver metabolism. Its expression is affected by epigenetic modifications such as DNA methylation, post-translational modifications, and microRNAs. Recently, HNF6 was also found to be expressed in tissues, such as the pancreas, intestine, and lungs, where it controls their formation by regulating cell differentiation and influences their pathophysiological processes via various mechanisms.

**Conclusion:**

In this review, we highlight advances in HNF6-related research concerning liver diseases and provide a summary of its potential mechanisms of action as a transcription factor in regulating downstream genes and epigenetic modifications. We also highlight gaps in liver disease research and provide future research directions for the application of HNF6 and its downstream molecules as attractive targets in the treatment of liver diseases.

## Background

The liver is an important and metabolically active organ involved in protein, carbohydrate, and lipid metabolism. Unhealthy lifestyle habits, oral medications, food poisoning, long-term heavy alcohol consumption, a high-fat diet, viral infection, and bacterial and parasitic infections can damage the liver, resulting in various liver diseases that pose a serious threat to human health (Delgado et al. 2021; Kawashima et al. 2024). Despite considerable progress in understanding the development of liver diseases, several challenges persist.

**Fig. 1:**
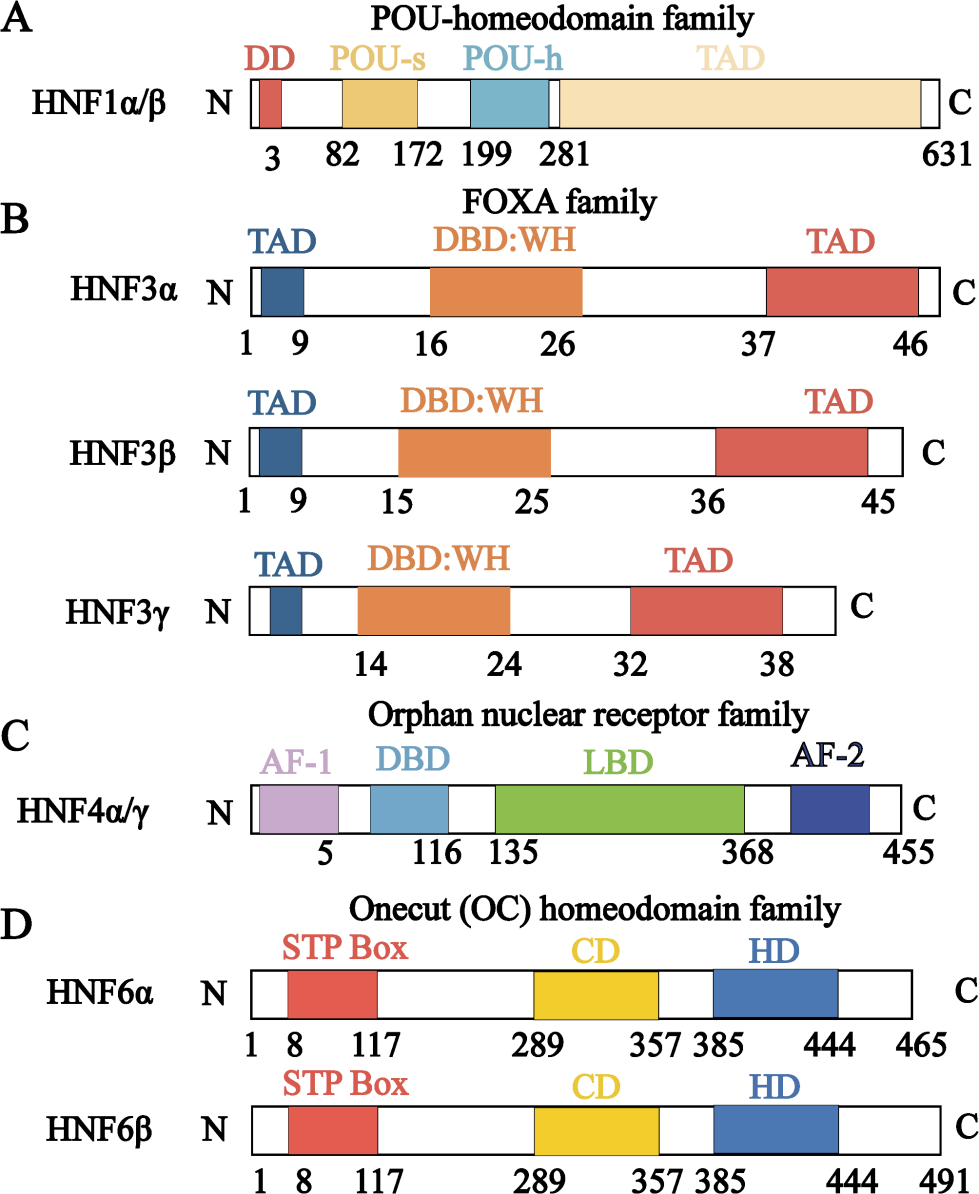
Structural domains of the HNF family. (**A**) Schematic structure of HNF1α/β. The N-terminus consists of a dimerization structural domain (DD), and the DNA-binding structural domain (DBD) consists of a POU-specific structural domain (POU-s) and a POU-homologous structural domain (POU-h). The C-terminus contains the transcriptional activation domain (TAD), and its full length consists of 631 amino acids. (**B**) Schematic structure of HNF3α/β/γ. HNF3 is a member of the forkhead box (FOX) protein subfamily, whose members include FOXA1, FOXA2, and FOXA3 (formerly known as HNF3α, HNF3β, and HNF3γ). Their structural domains contain a forkhead structural domain, also known as the winged helix (WH) structural domain, with a conserved TAD at both ends. WH is a DBD and the flanking sequences are essential for nuclear localization. (**C**) Schematic structure of HNF4 The orphan nuclear receptor family HNF4 consists of HNF4α and HNF4γ, which contain activation function domain 1 (AF-1) and a highly conserved DBD at the N-terminal end, and a ligand-binding structural domain (LBD) at the C-terminal end. The LBD also contains another activation function domain 2 (AF-2). (**D**) HNF6 (OC1) is part of the ONEGUT (OC) gene family, which also includes two homologous genes, OC2 and OC3. A single cleavage domain (CD) and a homology domain (HD) at the C-terminal end of the OC proteins together form the DBD, which mediates nuclear localization and transcriptional activation of the sequence together with the N-terminal STPbox (serine/threonine/proline-rich region)

Hepatocyte nuclear factors (HNFs) constitute a class of transcription factors that bind to hepatocyte-specific gene promoter regions and regulate the expression of these genes (Begum 2020; Toriyama et al. 2024). Either individually or via interactions, they form complex regulatory networks that precisely control the development and functioning of various tissues and organs, including the liver (Lau et al. 2018). Members of this family of transcription factors include HNF1, HNF3 (FOXA), HNF4, and HNF6 (ONECUT), which are expressed in different cells (Tachmatzidi et al. 2021). Specifically, HNF1α expression is significantly higher in hepatocytes from livers with drug-induced liver injury than in control livers (Zhang et al. 2021). Further, in normal adult livers, HNF3β is only expressed in biliary epithelial cells (BECs); however, under biliary cirrhosis and chronic biliary obstruction conditions, it is expressed in hepatocytes (Toriyama et al. 2024). Although HNF4α and HNF6 are predominantly expressed in normal adult livers, massive hepatic necrosis and chronic hepatitis C virus infection also induce their expression in BECs (Toriyama et al. 2024). HNF expression levels and distribution in the liver can be altered by pathological changes. Furthermore, the four HNFs have distinct molecular structures and functional domains (Fig. 1). However, they can be involved in crosstalk and interact with each other to form complex networks that collaboratively maintain liver homeostasis.

Particularly, HNF6 is predominantly expressed in the liver, and its functioning is more closely related to liver metabolism than that of the other HNFs. Notably, HNF6 is a transcriptional activator of genes that regulate bile homeostasis, glucose transport, and lipid metabolism (Russ-Silsby et al. 2023). It also regulates a complex network associated with several important biological functions in the liver. HNF6 also participates in the pathophysiological processes of various liver diseases. Thus, clarifying the role of HNF6 as well as its predecessors may facilitate the treatment of liver diseases.

In this study, we review the potential mechanisms of action of HNF6 as a key nuclear factor-regulated gene that is modulated by epigenetic modifications in the liver. We also explore its association with hepatic metabolic processes (i.e., liver glucose, lipid, and bile acid metabolism) and its roles in pathophysiological processes, including hepatitis, hepatic fibrosis, oncogenesis, and hepatic regeneration.

## HNF6

### HNF6 structure

In 1996, HNF6 was identified as the sixth member of the HNF family in mammals (Begum 2020). Although it was later observed that its components are similar to those of HNF5/HNF3 and HNF2/HNF4, the name HNF6 was retained (Begum 2020). The cleavage and homology domains of HNF6 form a DNA-binding region at the C-terminal end of the ONECUT protein, which, together with the N-terminal STPbox (serine/threonine/proline-rich region), mediate HNF6 nuclear localization and transcriptional activation (Tachmatzidi et al. 2021). Further, in hepatocytes, the HNF6 DNA-binding region binds to specific target gene sequences and regulates their transcription, a process that is essential for liver function (Tomaz et al. 2022). Cloning and sequencing the 8q24–q31 band gene of the rat chromosome indicated that HNF6 is produced via the selective splicing of three exons. These exons are more than 10 kb apart, with exon 1 encoding the N-terminal and shear domains, exon 2 encoding 26 HNF6β-specific amino acids, and exon 3 encoding the homology domain and C-terminal. Hence, HNF-6 has two isoforms, HNF6α and HNF6β with 465 and 491 residues, respectively, depending on the presence or absence of the 26 residues located between the cleavage and homology domains (Sheng et al. 2004).

### HNF6 function

HNF6 is involved in several crucial biological processes in the liver; it regulates cell growth by modulating the cell cycle (Peng et al. 2020). During liver tissue regeneration, it can stimulate the expression of cell cycle proteins, DNA synthesis, and mitosis-specific genes, promoting hepatocyte proliferation (Wang et al. 2008). Additionally, it is implicated in cell differentiation and organ formation (Sun et al. 2016) and plays a vital role in liver metabolism (Tachmatzidi et al. 2021). HNF6 also inhibits hepatitis B virus (HBV) gene expression and DNA replication via transcriptional and post-transcriptional pathways, thereby inhibiting hepatitis progression (Hao et al. 2015). One study indicated that the expression levels of transforming growth factor β1 (TGF-β1) and TGF-β receptor 2 (TGFBR2) are upregulated in the embryonic livers of HNF6-deficient mice, suggesting that HNF6 may be closely related to liver fibrosis (Tachmatzidi et al. 2021). Another study revealed decreased HNF6 expression in metastatic lesions of hepatocellular carcinoma (HCC), and its overexpression demonstrated that it functions as an anti-oncogene, inhibiting cancer cell migration and adhesion(Tachmatzidi et al. 2021). HNF6 also plays a role in some metastatic cancers, such as colon cancer, pancreatic cancer, lung adenocarcinoma, and bile duct cancer (Toriyama et al. 2024). Overall, it regulates a complex network that controls cell proliferation, cell cycle regulation, cell differentiation and organogenesis, cell migration, cell-matrix adhesion, glucose metabolism, tumorigenesis, hepatitis, and liver regeneration.

### HNF6 regulatory mechanisms

In addition to being an important organ for carbohydrate, protein, and lipid metabolism in the human body, the liver is also the production and storage site for various coagulation factors and is primarily responsible for the metabolic regulatory role of biological rhythms (Lange et al. 2024). Several studies have suggested that HNF6 can regulate the expression of genes related to these functions via direct or indirect pathways; we discuss these pathways below.

#### HNF6 directly regulates gene expression

HNF6 mediates the transcription of several hepatocyte-enriched genes, whose expression is required to maintain normal liver function. Initial studies revealed that HNF6 binds to several genes, including α-2-Macroglobulin (α-MG), α1-Antitrypsin (α-AAT), 6-phosphofructokinase-2 (PFKFB), as well as to major urinary proteins (MUPs), tryptophan oxygenase, and α-fetoprotein in the liver. It also binds to intestinal sucrase-isomaltase, homology domain proteins, and intestinal fatty acid-binding proteins (Samadani and Costa 1996). Further, it directly binds to gene promoter regions to regulate their expression in the liver, and consequently, regulates their related pathophysiological processes. For example, HNF6 mediates the regulation of important related proteins in pathophysiological processes. Additionally, protein C is a vitamin K-dependent zymogen of serine proteases that inhibits coagulation via the inactivation of the hydrolysis of Va and VIIIa proteins (Tripodi 2022). One study demonstrated that HNF6 is a major determinant of protein C gene activity, and that in venous thrombosis, the disruption of the binding site of HNF6 is primarily responsible for protein C deficiency (Spek and Reitsma 1999). Furthermore, fibroblast growth factor 21 (FGF21), a metabolic hormone that is expressed in the liver and regulates glucose metabolism and lipid homeostasis is closely associated with metabolism-related diseases, such as obesity, diabetes mellitus, and nonalcoholic fatty liver disease (NAFLD)(Geng et al. 2020). HNF6 can also regulate FGF21 expression, thereby affecting metabolic processes (Chavan et al. 2017), and it is also closely related to metabolic diseases.

As previously noted, HNF6 contains a single cleavage structural domain and a variable homology structural domain, both of which are required for its functioning as a gene transcription factor. The target sequences to which HNF6 binds dictate at least two modes of HNF6-DNA binding and two modes of transcriptional stimulation. Specifically, the conserved motifs of the homology structural domain of HNF6 bind to DNA monomers to activate target genes, and this part of the transcriptional stimulation is associated with F48M50 dichotomy. Further, the cleavage structural domain of HNF6, which is designed with an LXXLL (where L is leucine and X is any amino acid) motif, binds to DNA. Once this binding to the target gene occurs during transcription, the coactivator CBP/P300 is recruited to form a transcriptional activation complex, enabling HNF6 to bind more readily to the promoter region, thereby enhancing gene expression. This recruitment results in a strong interaction that depends on the LXXLL motif of the cleavage structural domain and the F48M50 dichotomy of the homology structural domain (Tachmatzidi et al. 2021). This observation provides evidence that HNF6 can regulate genes by binding to DNA via different regions.

#### Interactions of HNF6 with other HNFs

HNF6 and other HNFs synergistically function in a network of liver-enriched transcription factors that regulate liver differentiation and morphogenesis. Recently, several studies have demonstrated various interactions between HNF6 and other HNFs, playing numerous roles in liver functions.

First, it has been demonstrated that synergy exists between HNF6 and HNF3, and HNF6 initially recognizes the 138–126 region of HNF3 (HNF3β) promoter to activate its transcription (Wang and Holterman 2012). The physical interactions between HNF6 and HNF3 are achieved via the recruitment of P300/CBP. Another study indicated no significant differences in the binding site percentage on the Glut2 promoter between HNF3-deficient and control livers. Moreover, the sites where HNF6 bound to other targets also showed no significant differences (Rubins et al. 2005).

HNF6 also suppresses FOXA1 (HNF3α) expression by inhibiting TGFβ signaling pathway activity, representing a novel interaction among liver-enriched transcription factors, in which one factor indirectly controls the other by regulating signaling pathway activity (Plumb-Rudewiez et al. 2004). CYP2C12 gene expression depends on growth hormone (GH) secretion, and HNF6 competes with HNF3 for the same site in the CYP2C12 promoter region. Thus, CYP2C12 gene transcription is synergistically regulated by GH through HNF6 and HNF3. One study demonstrated that in liver samples from male rats, GH represses CYP2C12 promoter transcriptional activation by HNF3β and HNF6 via the activation of STAT5b. Thus, the female-specific expression pattern of CYP2C12 reflects the positive synergistic effects of GH-regulated transcription factors HNF3β and HNF6 (Delesque-Touchard et al. 2000).

Further, HNF6 synergizes with HNF4. The HNF4 gene has two promoter regions, proximal P1 and distal P2, which produce HNF4α1 and HNF4α7 transcripts, respectively, with the HNF4α7 transcript showing a lower expression level than the *HNF4α1* transcript throughout liver development (Beinsteiner et al. 2023). HNF6 also synergistically activates the P2 promoter with HNF1 in the embryo, resulting in increased HNF4α1 expression owing to the P1 promoter activity switch in the adult liver (Briançon et al. 2004). Transthyretin is an acute-phase protein in the liver, and HNF6 and HNF4 act synergistically to regulate its expression, decreasing its serum levels during the acute phase. While mutations in the binding site of HNF4 to the transthyretin gene promoter do not affect HNF6-HNF4α interactions (Wang and Burke 2010), HNF6 and HNF4 interactions remain a new area for future research.

HNF6 also interacts with HNF1, and both synergistically stimulate protein C expression. They also have completely overlapped binding sites in the protein C gene promoter region, which plays a key role in coagulation. The disruption of these binding sites results in protein C deficiency, as well as an increased risk of venous thrombosis (Spek and Reitsma 1999). During the directed differentiation of human pluripotent stem cells (hPSCs) into hepatocyte-like cells (HLCs), the simultaneous overexpression of HNF1α, HNF6, or Foxa3 results in features characteristic of mature hepatocytes, including CYP3A4 activity, protein secretion, and hepatotoxic responses (Tomaz et al. 2022). Overall, these results suggest that HNFs interact with each other for synergistic transcriptional activation and these interactions play important roles in maintaining tissue-specific gene expression.

#### HNF6 expression is regulated by epigenetic modifications

Sex differences in hepatic gene expression may be responsible for liver diseases and may increase the risk of thrombosis, dyslipidemia, coronary artery disease, and various liver diseases. Chronic hepatitis, primary sclerosing cholangitis, HCC, NAFLD, steatohepatitis, and hepatic fibrosis are more common in male individuals, whereas primary biliary cirrhosis, autoimmune hepatitis, and alcoholic hepatitis are more prevalent in female individuals (Tachmatzidi et al. 2021; Wang et al. 2021). HNF6 is a sex-dependent, GH-regulated transcription factor; it is expressed at a 2.80-fold higher level in female individuals than in male individuals; however, the methylation levels of its promoter are similar in both sexes. When dopamine D2 receptors in neurons are disrupted, the expression levels of GH-releasing hormone (GHRH) in the hypothalamus, GH in the pituitary gland, and insulin-like growth factor 1 (IGF-1) in serum are significantly reduced. This leads to increased HNF6 promoter methylation, which subsequently significantly downregulates HNF6 expression in female individuals, whereas no such changes are observed in male individuals. Moreover, decreases in HNF6 expression levels are negatively associated with the methylation of its promoter (Brie et al. 2020). Consistent with previously reported findings, the proximal promoter and exon of the HNF6 gene, with binding sites for STAT5b, C/EBPα, SP1, and HNF4, are characterized by larger CpG islands (Brie et al. 2020). Further, it has been suggested that HNF6 expression is involved in post-translation modification regulation. Another study demonstrated that CBP/P300 promotes acetylation modification at the K339 and L330 sites of HNF6, thereby enhancing HNF6 protein stability. However, owing to the proteasomal degradation pathway, mutations in K339R do not result in HNF6 instability (Rausa et al. 2004). Protein kinase A (PKA) can phosphorylate HNF6 to upregulate glucose-6-phosphatase (G6Pase) fusion gene expression in the HCC cell line, HepG2 (Streeper et al. 2001). Additionally, miRNAs act on mRNA to regulate HNF6 gene expression, and miR-495 upregulates endogenous HNF6 expression (Prévot et al. 2013). In contrast, miR-Let-7b in mesenchymal stem cells downregulates HNF6 expression (Alizadeh et al. 2015).

**Fig. 2:**
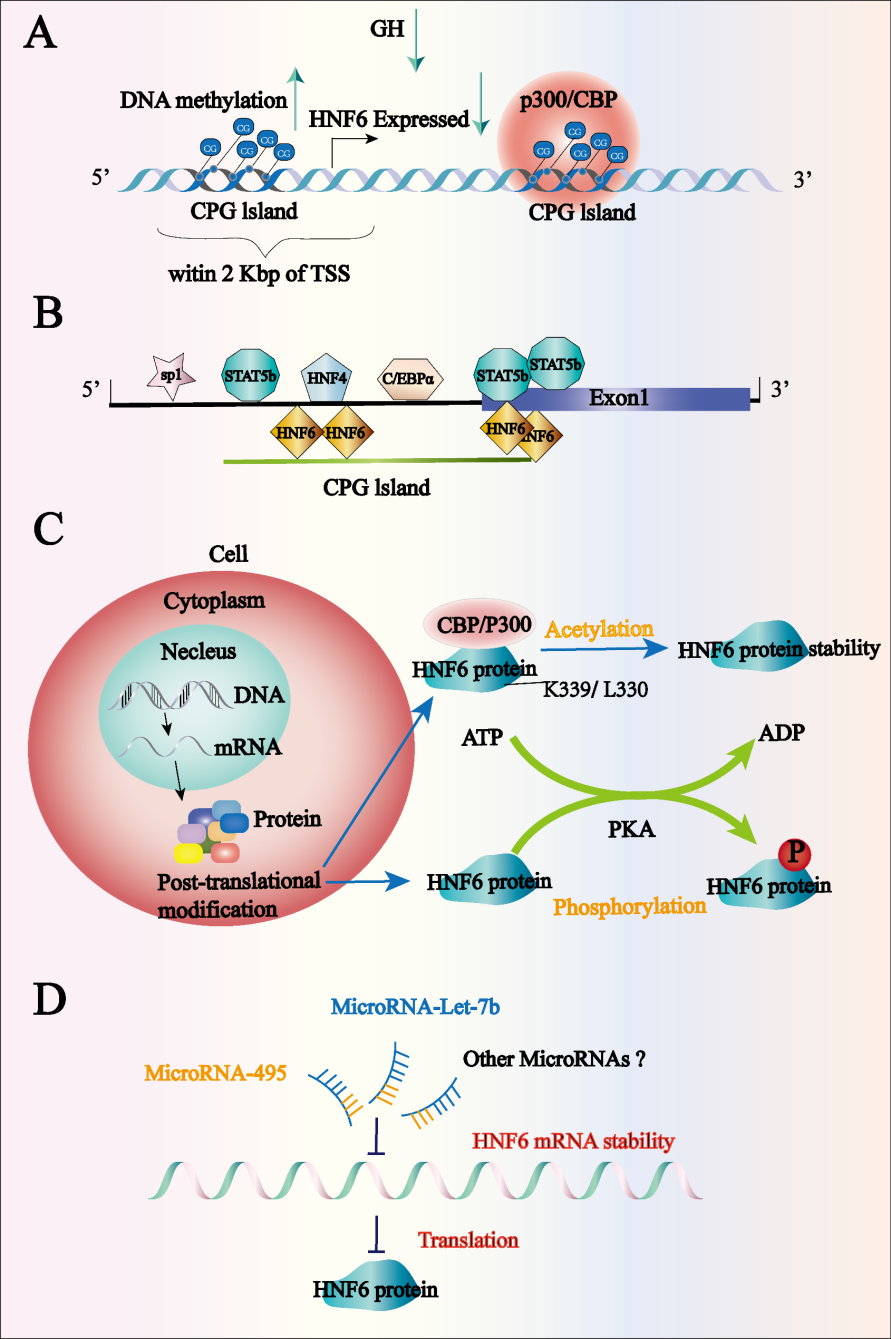
HNF6 expression is regulated by epigenetic modifications. (**A**) Elevated levels of DNA methylation in the HNF6 promoter region result in a significant downregulation of HNF6 expression in female individuals but not in male individuals. Moreover, HNF6 is a sex-dependent transcription factor regulated by growth hormone (GH). (**B**) Binding sites for STAT5b, C/EBPα, SP1, and HNF4 are present in the CpG island in the proximal promoter region of the HNF6 gene. (**C**) HNF6 expression is involved in the regulation of post-translational modifications; CBP/P300 promotes acetylation modifications at the HNF6 K339 and L330 sites, thereby enhancing HNF6 protein stability. Protein kinase (PKA) phosphorylates HNF6 to function. (**D**) MiRNAs act on mRNAs to regulate *HNF6* gene expression, such as miR-495 acting to upregulate endogenous HNF6 expression, whereas miR-Let-7b downregulates HNF6 expression

In summary, HNF6 expression is regulated by epigenetic modifications (Fig. 2). Other undiscovered epigenetic regulatory mechanisms may exist, representing promising research directions. Thus, further studies are required in this regard.

## HNF6 in liver metabolism

**Fig. 3:**
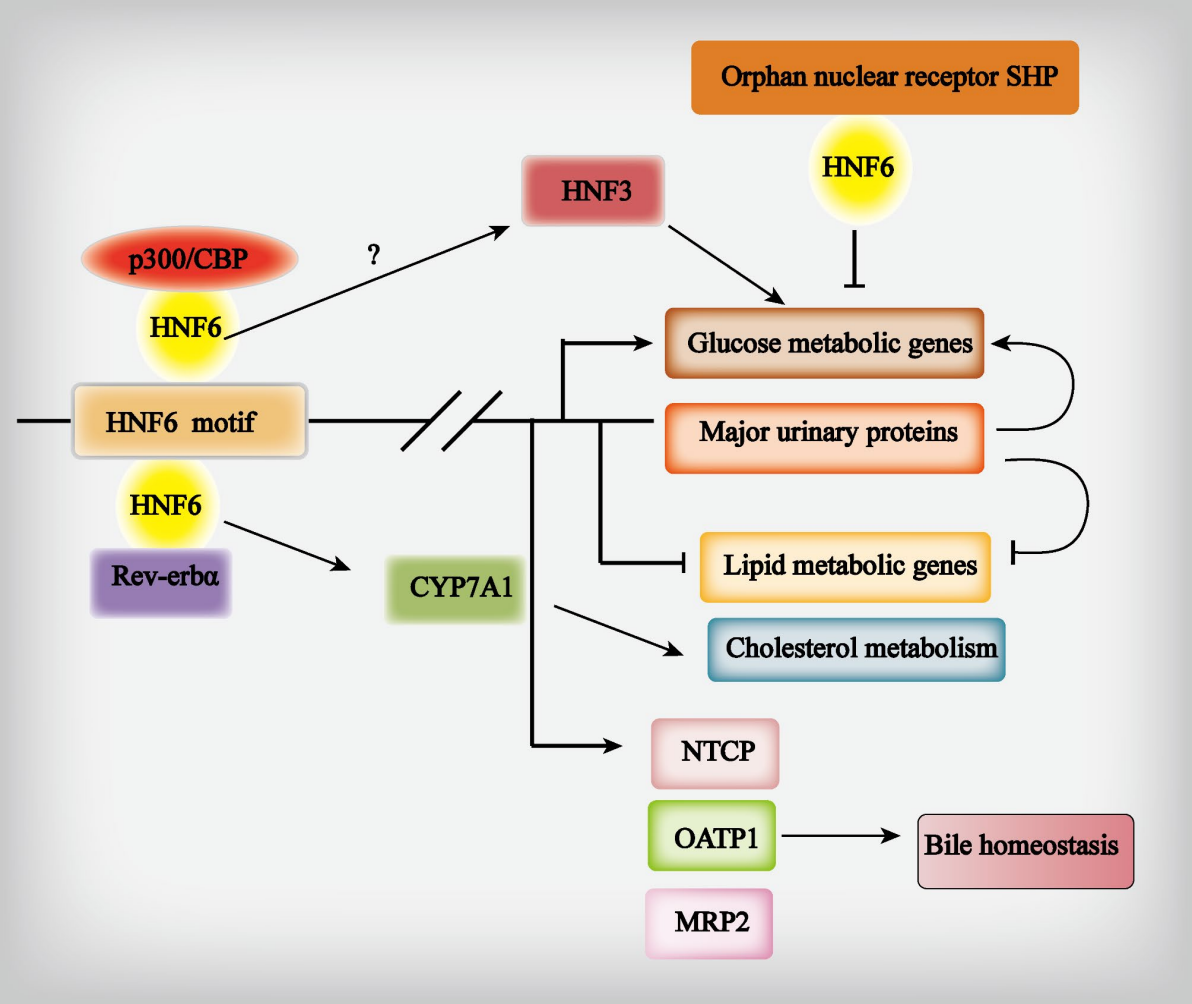
HNF6 is involved in the regulation of hepatic glucose metabolism, lipid metabolism, and bile acid metabolism. (**1**) Role of HNF6 in glucose metabolism. CBP/P300 is essential to enhance the ability of HNF6 to respond to the transcription of glucose metabolism-related genes. HNF6 directly regulates the expression of glucose genes involved in glucose metabolism. HNF6 regulates glucose metabolism through major urinary protein (MUP). HNF6 regulates glucose metabolism through MUP. This regulatory pathway may serve as a new mechanism by which MUP acts as an intermediate molecule to indirectly regulate glucose. HNF6 may regulate glucose metabolism-related enzyme synthesis through HNF3. Small heterodimer partner (SHP), an atypical orphan nuclear receptor, is a new target for HNF6 to regulate glucose metabolism-related gene production. (**2**) Role of HNF6 in lipid metabolism. HNF6 regulates the expression of the CYP7A1 gene and is involved in cholesterol metabolism. HNF6 and Rev-erbα coordinate to regulate hepatic lipid metabolism. MUP regulates lipid metabolism by repressing the expression of lipogenic genes. (**3**) Role of HNF6 in bile homeostasis. HNF6 controls bile acid metabolism through the regulation of sodium taurocholate cotransporter protein (NTCP), organic anion transporting polypeptide 1 (OATP1), and multidrug resistance-associated protein 2 (MRP2)

HNF6 regulates enzymes involved in carbohydrate, fat, and bile acid metabolism in the liver. It is also implicated in glucose level regulation, lipid metabolism, and bile acid biosynthesis (Fig. 3).

### Role of HNF6 in glucose metabolism

HNF6 can regulate glucose metabolism and levels in the circulatory system via various mechanisms. G6Pase plays a crucial role in gluconeogenesis, and mutational analysis of the G6Pase promoter revealed that its promoter region binds to HNF6. Further, inhibiting HNF6 binding to the G6Pase promoter region abolishes the PKA stimulatory effect of HNF6 on G6Pase(Streeper et al. 2001). Notably, PKA phosphorylates HNF6 i*n vitro*. Hence, after phosphorylation by PKA, HNF6 can activate G6Pase fusion gene expression. A previous study indicated that *HNF3* overexpression decreases the levels of hepatic glycogen, glucose transporter protein 2 (Glut-2), and HNF6. However, co-overexpressing HNF3 and HNF6 upregulates hepatic glycogen and Glut-2 levels in mouse livers, whereas HNF6 overexpression alone results in a two-fold increase in hepatic Glut-2 levels, indicating that HNF6 triggers Glut-2 transcription in vivo. Further analyses in mice have revealed that only recombinant HNF6 protein, rather than recombinant HNF3 protein, binds to the 185–144-bp Glut-2 promoter sequence (Tan et al. 2002). HNF6 controls glucose absorption by transcriptionally activating Glut-2 expression. Additionally, the acetylation of the transcriptional coactivator CBP is essential for HNF6 protein stability and for enhancing the ability of HNF6 to respond to Glut-2 promoter transcription (Rausa et al. 2004).

Glucokinase plays a key role in glucose metabolic homeostasis. HNF6 can influence diabetes progression by targeting glucokinase to control glucose metabolic homeostasis. Further, HNF6-deficient mice exhibit impaired glucose-stimulated insulin secretion and ultimately develop diabetes (Jacquemin et al. 2000). Therefore, HNF6 may be a candidate gene for diabetes treatment. However, in this previous study, 20–25% of *HNF6*-deficient mice did not develop diabetes. Lannoy et al. demonstrated that in the liver, HNF6 binds to the glucokinase gene promoter, whereas no such binding occurs in the pancreas. Further, mutations in the binding site for HNF6 in the glucokinase gene may lead to diabetes; however, these mutations are unrelated to glucose tolerance (Tachmatzidi et al. 2021). This observation may partially explain the diabetes observed in *HNF6*-knockout mice, i.e., diabetes in *HNF6*-deficient mice may be caused by functional defects in both the pancreas and liver. HNF6 is known to regulate the expression of some MUPs in mice, particularly MUP1 (Samadani and Costa 1996). Studies have also shown that MUP1, which regulates glucose metabolism by suppressing gluconeogenic gene expression, is significantly downregulated in hereditary and dietary diabetes mice. However, HNF6 may regulate glucose metabolism via MUP1; this regulatory pathway potentially represents an indirect novel mechanism of glucose regulation by MUP1, acting as an intermediate molecule (Zhang et al. 2024a, b). HNF3 provides another indirect pathway by which HNF6 regulates glucose metabolism. Elevated HNF3 levels affect the expression of glucose metabolism-related enzymes, such as 6-phosphofructokinase-2 and G6Pase, which are involved in glucose homeostasis (Lemaigre et al. 1993). In one study, HNF3-overexpressed transgenic mice exhibited depleted hepatocyte glycogen stores, which were associated with reduced expression of phosphoenolpyruvate carboxylase (PEPC) and glycogen synthase (Wu et al. 2016). Moreover, HNF3 transcriptional activation was found to always involve HNF6. HNF6 may regulate the synthesis of these carbohydrate metabolism-related enzymes via HNF3. It also antagonizes glucocorticoid-stimulated gene transcription and expression. For example, HNF6 inhibits the expression of two key enzymes, PEPC and 6-phosphofructokinase-2, in glucocorticoid-induced glucose metabolism (Tachmatzidi et al. 2021). Small heterodimer partner (SHP) is an atypical orphan nuclear receptor that functions as a transcriptional repressor via its interaction with diverse nuclear receptors and transcription factors. SHP has also been shown to not only inhibit HNF6 transcriptional activity but also inhibit G6Pase and PEPC expression via HNF6 (Lee et al. 2008). Therefore, SHP may be a novel target through which HNF6 regulates gluconeogenesis.

### Role of HNF6 in lipid metabolism

Tissue-specific regulatory circuits formed by HNF6 and other transcription factors act as key regulators of transcription in hepatocytes and participate in lipid metabolism via different mechanisms, in addition to their involvement in glucose metabolism. The CYP7A1 gene encodes cholesterol 7α-hydroxylase, an enzyme that catalyzes cholesterol catabolism. CYP7A1 deficiency causes hypercholesterolemia (Zhang et al. 2024a, b), and during bile duct ligation (BDL), both HNF6 and CYP7A1 are significantly downregulated, resulting in a severe disruption of cholesterol catabolism. Co-transfection and gel migration assays have confirmed that HNF6 transcriptionally regulates CYP7A1 gene expression in mice liver and HepG2 cells (Wang et al. 2004). Further, HNF6 deletion in normal chow-fed C57BL/6 mice results in hepatic steatosis owing to the upregulation of several lipid metabolism genes, most of which are targets of Rev-erbα, a circadian nuclear receptor that connects the biological clock to hepatic metabolism. Moreover, HNF6 deletion specifically disrupts Rev-erbα binding at these loci (Zhang et al. 2016). HNF6 and Rev-erbα coordinately regulate hepatic lipid metabolism. HNF6 also acts as an activator of other genes to regulate lipid metabolism. For example, *MUP1*, an HNF6-regulated gene, is significantly downregulated in genetic and diet-induced obesity. MUP1 also regulates lipid metabolism by suppressing lipogenic gene expression (Zhang et al. 2024a, b). Additionally, increased HNF3 expression alters exosome composition in intestinal epithelial cells to prevent insulin resistance in high-fat diet-fed mice (Kumar et al. 2022). Promethazine activates the HNF3 pathway, ameliorating steatosis (Liu et al. 2022). These findings indicate that HNF6 can be activated via HNF3 pathways that regulate lipid metabolism.

### Role of HNF6 in bile homeostasis

During embryonic development, hepatocytes differentiate into BECs, which gradually proliferate and differentiate, eventually forming the complete bile duct system. This system gradually branches to form a biliary dendritic structure and eventually a biliary network. BECs regulate the uptake and excretion of water, electrolytes, and organic substances from bile through various intracellular ion channels and transporter proteins. Collectively, these processes maintain bile secretion and formation. Sodium taurocholate co-transporter protein (NTCP) is primarily responsible for bile acid transport in the liver (Salhab et al. 2022). However, in humans, mice, and rats, NTCP is regulated by HNF6, which is also essential for the early differentiation of hepatocytes into BECs. HNF6-deficient mice develop bile duct malformations as well as cholestasis-induced death. Further, HNF6 is highly expressed in BECs of mature mouse livers and negatively regulates BEC proliferation (Holterman et al. 2002). It may also regulate the expression of the transporter genes involved in bile synthesis. The expression levels of bile transporter genes, organic anion transporting polypeptide 1 (OATP1), NTCP, and multidrug resistance-associated protein 2 (MRP2) are reportedly increased in cell lines overexpressing HNF6 (Glaser et al. 2010). OATP1 is an uptake transporter protein responsible for transporting various endogenous and exogenous substances, including bile acids and steroid hormones, into hepatocytes (Campagno et al. 2021). OATP1 expression is severely reduced in hepatocyte-specific mice with HNF6 deletion, suggesting that it serves as a target gene for HNF6 (Glaser et al. 2010). Hepatocyte damage and apoptosis induced by bile acids are attenuated in *Stat5*-deficient mice owing to GH-induced downregulation of hepatoprotective genes, HNF6 and OATP1, consistent with findings based on OATP1 deletion (Blaas et al. 2010).

When bile acids are excreted into the small intestine and reabsorbed, approximately 80% of them are transported to hepatocytes via NTCP and subsequently secreted into bile to form the enterohepatic bile acid cycle (Park et al. 2022). MRP2 is primarily expressed in the basement membrane of human hepatocytes and is an important driver of bile flow; it promotes bile secretion and increases bile acid salt lipid solubility. However, no enhanced expression of whole liver NTCP or MRP2 has been observed in these HNF6-regulated genes (Tachmatzidi et al. 2021). This suggests that the expression of these two genes cannot be completely attributed to the HNF6 transcriptional action on their promoters.

Additionally, HNF6 affects bile homeostasis by regulating HNF3, which can disrupt bile homeostasis via various pathways, including the endoplasmic reticulum stress pathway (Bochkis et al. 2008), which is required for bile biosynthesis and transport in the liver (Aghadi et al. 2022).

Collectively, the overlapping functions and different mechanisms of action of HNF6 in glucose metabolism, lipid metabolism, and bile homeostasis warrant further investigation to fully clarify their roles in glucose-lipid metabolism or other hepatic metabolic processes.

## Involvement of HNF6 in abnormal pathophysiologic processes in the liver

**Fig. 4:**
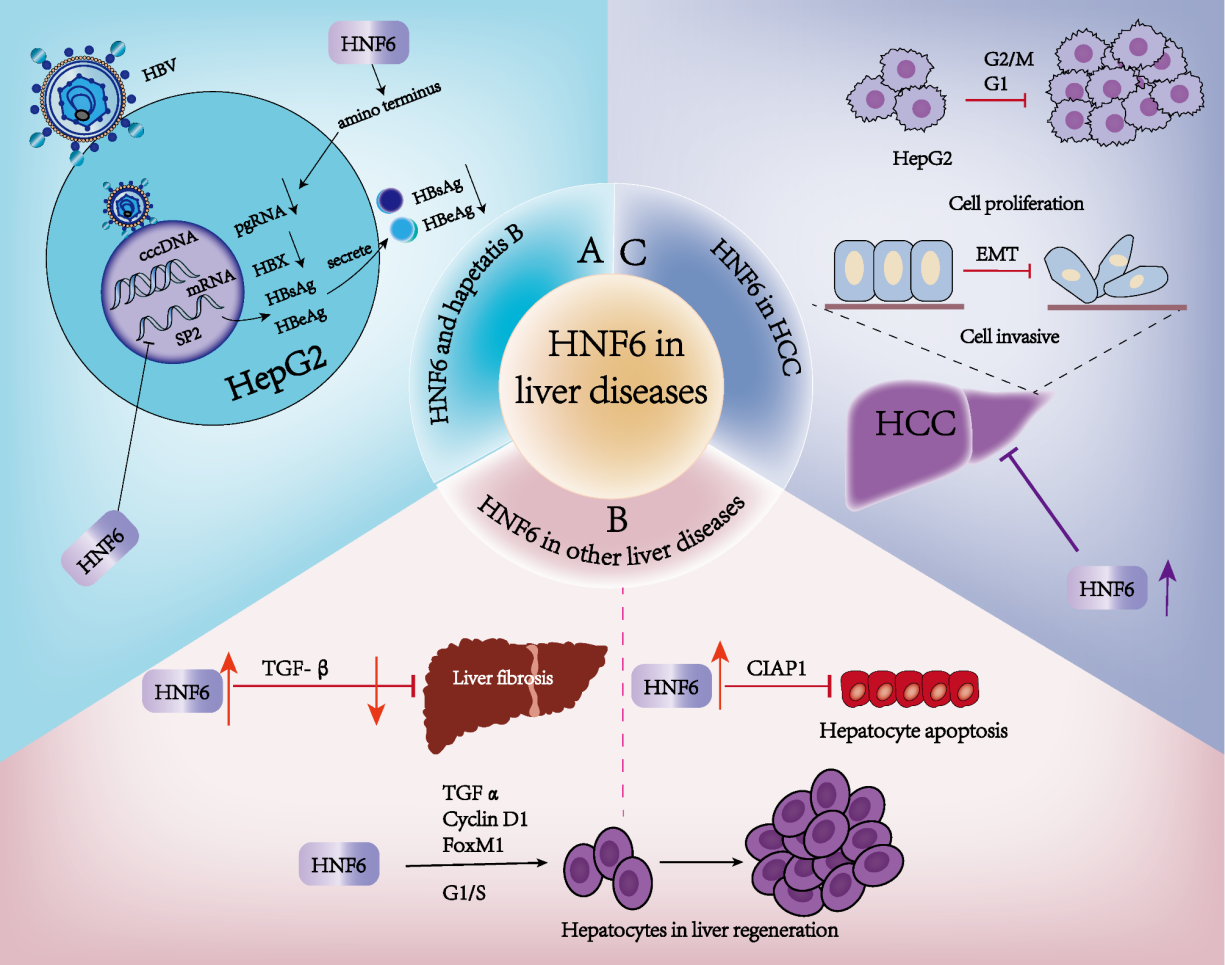
HNF6 is involved in abnormal pathophysiological processes in the liver, such as liver injury, fibrosis, and tumorigenesis. (**A**) HNF6 inhibits HBV gene expression and DNA replication by repressing SP2 transcription, thereby reducing HBV RNA levels and secreted viral proteins in extracellular media. Moreover, this inhibitory effect is mainly attributed to the N-terminus of HNF6. (**B**) During BDL-induced liver injury, upregulation of HNF6 reduces the expression of the pro-fibrotic factor TGF-β and decreases fibrotic injury. Additionally, HNF6 targets the expression of inhibitor of apoptosis protein 1 (IAP1), a process that is accompanied by a decrease in hepatocyte apoptosis. HNF6 promotes the transcriptional activity of transforming growth factor α (TGFα), cyclin D1, and forkhead box m1 (FoxM1) transcription factors, controlling the progression of the S phase, which plays an important role in the regeneration of hepatocytes. **(C**) In hepatocellular carcinoma (HCC), HNF6 overexpression inhibits the proliferation of HCC cells by arresting the cell cycle of HepG2 cells in the G2/M phase. In addition, increased HNF6 expression upregulates the expression of differentiation-related markers and activates epithelial-mesenchymal transition (EMT), which promotes hepatocyte differentiation and inhibits cell migration and invasion

All liver diseases pose a serious threat to human health. HNF6 is reportedly involved in abnormal pathophysiological processes in the liver, such as liver injury, fibrosis, and tumorigenesis (Fig. 4).

### Liver injury

HBV (approximately 3.2 kb in size) is a DNA virus that is partially double-stranded, loosely circular and tends to target the liver. According to data from the World Health Organization, approximately 2 billion people worldwide are infected with HBV and approximately 780,000 people die each year from HBV-related liver failure, cirrhosis, and HCC (Iannacone and Guidotti 2022). Recent studies have demonstrated that HNF6 inhibits HBV gene expression and DNA replication(Hao et al. 2015). Further, HNF6 overexpression significantly reduces HBV RNA levels and secreted viral proteins in the extracellular medium, and a stronger inhibitory effect has been observed on HBsAg than on HBeAg. When short hairpin RNA was used to decrease HNF6 expression in HepG2 cells, the expression levels of HBV RNA, HBV DNA, and secreted HBsAg and HBeAg increased significantly compared to their levels in the controls. These findings indicate that HNF6 inhibits HBV gene expression and replication. Further, mechanistic analyses indicated that a cis-element in the HBV genome (nt 3009–3019) is responsible for SP2 promoter repression by HNF6. Moreover, HNF6 decreased viral genomic pregenomic RNA expression at the post-transcription level, with the N-terminus of HNF6 being primarily responsible for mediating this repressive effect. Hydrodynamic injection of pHBV1.3 and HNF6 expression plasmids or their control vectors into mice significantly reduces HBV gene expression and DNA replication (Hao et al. 2015). HNF6 is a host factor that restricts HBV replication via transcriptional and post-transcriptional mechanisms, suggesting that it is a potential therapeutic target in HBV-associated hepatitis-induced liver injury.

With the development of liver transplantation techniques, liver repair and regeneration have been extensively studied. Liver regeneration is a complex process, and related studies have demonstrated the involvement of HNF6 in this process. During liver regeneration after partial hepatectomy in mice, HNF6 adenovirus injection via the tail vein significantly increases the number of hepatocytes entering DNA replication (S phase) owing to elevated HNF6 levels. Additionally, hepatic macrophages stimulate HNF6 expression in hepatocytes and promote hepatocyte proliferation during early liver regeneration stages (Peng et al. 2020). Chromatin immunoprecipitation analysis of hepatocytes during liver regeneration after partial hepatectomy in mice revealed that HNF6 promotes the transcriptional activity of transforming growth factor alpha (TGFα), cell cycle protein D1 (Cyclin D1), and forkhead box m1 (FoxM1) transcription factors, thereby regulating the progression of S phase. Notably, BDL liver-injured mice exhibit significantly downregulated HNF6 expression and reduced cell proliferation. However, the treatment of these mice with adenovirus overexpressing HNF6 or GH upregulates HNF6 expression and cell proliferation, thereby attenuating BDL-induced liver injury (Wang et al. 2008).

### Liver fibrosis

The activation of hepatic stellate cells (HSCs) is a major driver of fiber formation in liver fibrosis. Further, the deposition of extracellular matrix components secreted by activated HSCs disrupts liver structure. When the liver is injured, the secretion of TGF-β, a pro-fibrotic cytokine, in the fibrotic microenvironment increases, resulting in the activation of downstream signaling pathways translocated to the nucleus via TGFBR1/2. This activation directly targets downstream genes, initiating the expression of extracellular matrix components. HNF6 also plays a role in liver fibrosis. Additionally, TGFBR2 expression and the TGFβ1 signaling pathway are upregulated in the embryonic livers of HNF6-deficient mice. During BDL-induced liver injury, GH upregulates HNF6 expression, which then upregulates the expression of Cyclin D1 and CYP7A1, while decreasing that of pro-fibrotic TGF-β. These changes in protein expression level result in improved liver function, including increased hepatocyte proliferation, enhanced cholesterol clearance, and reduced fibrotic injury (Wang et al. 2008). HNF6 expression is significantly downregulated in the livers of rats with 10 weeks of HLCs.

choline deficiency diet-induced nonalcoholic steatohepatitis and advanced liver fibrosis (Mu et al. 2009). Overall, the GH/HNF6 axis represents a potential in vivo mechanism that regulates hepatic repair responses during biliary tract injury in mice.

Liver fibrosis and cirrhosis development are often accompanied by GH resistance and low IGF-1 levels. A mouse model of inflammatory cholestasis and hepatic fibrosis in Ghr-knockout mice crossed with multidrug resistance gene 2-knockout mice exhibit downregulated HNF6, IGF-1, and STAT5 expression levels. HNF6 expression in this liver fibrosis model may provide a strategy for liver fibrosis treatment (Blaas et al. 2010). Further, in a model of BDL-induced liver injury, specific HNF6 knockdown in hepatocytes increased hepatocyte apoptosis and fibrotic injury. In contrast, in primary hepatocytes from BDL-induced mice, co-treatment with GH/lipopolysaccharide enhanced HNF6 expression in hepatocytes. HNF6 also targets and regulates the expression of inhibitor of apoptosis 1 protein (IAP1), and this process is accompanied by decreased hepatocyte apoptosis (Wang et al. 2016). These findings suggest that HNF6 can inhibit hepatocyte apoptosis and fibrotic injury by targeting IAP1. Further, fibrotic injury may be mitigated via TGFβ signaling pathway inhibition or other mechanisms.

### Role of HNF6 in tumorigenesis

Carcinogenesis is closely related to tumor cell proliferation, differentiation, migration, and adhesion. In HepG2 cells, HNF3 knockdown restores HNF6 activity, leading to a multifold increase in the expression of HNF6-targeted genes. In a previous study, a BrdU labeling assay revealed that HepG2 cells with HNF6 overexpression exhibit a 50% decrease in cell proliferation rate, indicating that HNF6 inhibits HCC cell growth. Another study demonstrated that HNF6 inhibits HCC cell proliferation by suppressing the cell cycle of HCC cells in the G2/M phase (Lehner et al. 2010). Further, the expression levels of HNF6 and Krüppel-like factor 4 (KLF4) are directly related, and reduced KLF4 or HNF6 expression levels are associated with a higher HCC grade. Poorly differentiated HCC cells show lower KLF4, HNF6, and differentiation-related marker expression levels than highly differentiated HCC cells. Mechanistically, *KLF4* transcription activates HNF6 expression, and this increased HNF6 expression upregulates the expression of differentiation-related markers to promote HCC cell differentiation and inhibit cell migration and invasion (Sun et al. 2016). HNF6 is also downregulated in cholangiocarcinoma, pancreatic ductal adenocarcinoma, and lung adenocarcinoma (Toriyama et al. 2024). Its overexpression in tumors can inhibit cell proliferation, migration, invasion, tumor cell de-differentiation, and tumor progression; therefore, targeting HNF6 could be a strategy for tumor treatment.

Notably, HNF6 is not expressed in primary colorectal cancer (CRC), but it is expressed in CRC liver metastasis. Additionally, HNF6 acetylation is critical for HNF6 transcriptional activity (Lehner et al. 2007). Lehner et al. observed that unacetylated HNF6 is significantly upregulated in colorectal liver metastases and in the CRC cell line, SW620-HNF6, which stably expresses HNF6. Transwell and scratch assays have also demonstrated that SW620 cells exhibited decreased migration, suggesting that HNF6 can inhibit the CRC liver metastasis process. Therefore, HNF6 may be responsible for tumor cell adhesion to liver tissue (Lehner et al. 2010). Restoring acetylated HNF6 and, thus, its DNA-binding activity may offer a therapeutic approach for preventing disease progression and inhibiting colorectal liver metastasis. However, in a recent study, contrasting results were obtained; HNF6 expression was found to be upregulated in both CRC primary sites and liver metastases, and its high expression level indicated poor survival in patients with CRC (Jiang et al. 2019). Therefore, the function of HNF6 in liver metastasis requires further investigation.

## Conclusions

Since the discovery of HNF6 in the late 20th century, numerous studies have demonstrated that it can function as a key nuclear factor in the liver. Specifically, it controls gene expression in the liver and thus affects various biological functions. The expression site, structure, and function of HNF6 are well understood. It exhibits a wide range of biological effects, and in this review, we have comprehensively summarized its role as an important regulatory factor in liver metabolism and in controlling various liver diseases. We have elaborated upon the direct and indirect modes of action of HNF6 with respect to gene expression regulation as well as the mechanisms through which it is regulated by different epigenetic modifications. HNF6 affects various metabolic pathways by regulating the expression of glucose, lipids, and bile acid metabolism-related genes, thereby affecting disease progression. We also noted that while the roles and mechanisms of action of HNF6 in diabetes have been thoroughly studied, the existing research findings are primarily based on in vitro cell models. Therefore, to translate these findings in clinical practice, verification using in vitro disease models and confirmation based on sufficient clinical data are necessary. Additionally, HNF6 regulates the expression of HBV-related secretory proteins and pregenomic RNAs via both transcriptional and non-transcriptional pathways. Thus, it demonstrates the potential to serve as a therapeutic target for HBV-related liver diseases. Additionally, HNF6 acts as a tumor suppressor in liver cancer and may also represent a new treatment strategy for different cancers. However, its regulatory mechanism in this regard requires further exploration. In summary, HNF6 is crucial for liver health, and thorough related research may provide a new direction for treating liver diseases.

## Data Availability

No datasets were generated or analysed during the current study.

## Abbreviations

αMG: Alpha macroglobulin
α-AAT: Alpha-1-antitrypsin
BDL: Bile duct ligation
CRC: Colorectal cancer
FGF21: Fibroblast growth factor 21
GH: Growth hormone
GHRH: GH-releasing hormone
Glut-2: Glucose transporter protein 2
G6Pase: Glucose-6-phosphatase
HCC: Hepatocellular carcinoma
HSCs: Hepatic stellate cells
hPSCs: Human pluripotent stem cells
KLF4: Krüppel-like factor 4
MUPs: Major urinary proteins
NTCP: Sodium taurocholate co-transporter protein
OATP1: Organic anion transporting polypeptide 1
PFKFB: 6-phosphofructokinase-2
PEPC: Phosphoenolpyruvate carboxylase
PKA: Protein kinase A
SHP: Small heterodimer partner
TGF-β1: Transforming growth factor β1
TGFBR2: TGF-β receptor 2
TGFα: Transforming growth factor alpha
